# Increase of vanillin partitioning using aqueous two phase system with promising nanoparticles

**DOI:** 10.1038/s41598-019-56120-8

**Published:** 2019-12-23

**Authors:** Mitra Nouri, Shahla Shahriari, Gholamreza Pazuki

**Affiliations:** 1 Department of Food Science and Technology, Shahr-e-Qods Branch, Islamic Azad University, Tehran, Iran; 2 Department of Chemical Engineering, Shahr-e-Qods Branch, Islamic Azad University, Tehran, Iran; 3 Department of Chemical Engineering, Amirkabir University of Technolgy (Tehran Polytechnic), Tehran, Iran

**Keywords:** Chemical engineering, Carbon nanotubes and fullerenes

## Abstract

The distinct features of ATPSs (aqueous two-phase systems) have made it possible to promote the extraction efficiency of biomolecules. The purpose of this study is to discover an appropriate nanoparticle to design an economical optimal separation process, and to understand the underlying molecular mechanism which allows the partitioning of vanillin as a phenolic compound using nanoparticle-based ATPSs. To this aim, the capabilities of several different nanoparticles were investigated as additives for boosting the partition coefficient of vanillin in two different ATPSs made up of polyethylene glycol and sodium sulfate/polyethylene glycol and dextran. Also, in an attempt to explain the salting-out effect, the NRTL (Non-random Two Liquid) thermodynamic model was applied. The impact of very small amounts of modified carbon nanotubes on the enhancement of the partition coefficient of vanillin in the ATPS consisting of the biocompatible polymer(s) and salt was quite remarkable. The results showed that the partition coefficient of vanillin grew by almost 127 percent compared to the system without nanoparticle. The molecular mechanism underlying the increase in the partition coefficient was interpreted by taking advantage of structural analyses.

## Introduction

The emergence of two-phase systems since 1956 and their extensive expansion in recent decades have proven the importance and worthiness of these systems for the separation of biomolecules.

In aqueous two-phase systems (ATPs), the extraction is due to the contact of two immersive liquids (colloidal system), which is related to the mass transfer operations. In this process, the system is initially well mixed and then will reach equilibrium from one phase to another phase little by little due to diffusion of components. In this method, the purpose is to transfer a component (vanillin) from one phase to other phases. The evaluation of the effectiveness of this method is on the basis of the division of the intended component between the two phases and its selection, which is determined by the partition coefficient.

One of the main purposes of the researchers is the selection of efficient and economically feasible conditions that cause the separation and purification of biomolecules in aqueous two-phase systems (ATPs) to improve. So far, many changes and developments have been made in ATPs to obtain the extraction efficiency^1,2^. But the thing that has played a key role in the development of an ATPS has been based on the changes in the concentration of the constituents, the system temperature, the system pH, the molecular weight of polymer, the hydrophobicity of salt, the type of salt, the ionic strength of the system, and the change of the system components (*e.g*. ionic liquids, carbohydrates, etc)^3^. The deep study and understanding of the simultaneous effects of the multiple contributing variables on the partition coefficient of biomolecules could be complicated. Although in some cases, presenting a fundamental analysis is not possible, the observed changes in the ATPSs are often interpreted based on error-guessing tests as well as the simulation of the behavior of similar systems.

Recently, a convenient novel approach has been proposed, which includes adding the nanoparticles as well as their significant capacity in the development of the partitioning of biomolecules in ATPSs^4–6^. In this method, it is assumed that the spontaneous partitioning of nanoparticles in ATPSs, in which the nanoparticles act as an absorbent, so that it causes the biomolecule to be transferred to one of the phases^6^. In this case, the main focus is on the binding of the nanoparticle to the target biomolecule. It should be mentioned that the strongest point in the strategy of introducing nanoparticles is the absence of the need for changing the structure of the system. Metal oxide nanoparticles have wide applications in various industries thanks to their low cost and simplicity in production. Iron oxide nanoparticles as an antibiotic carrier^7^, and also titanium dioxide and zinc oxide nanoparticles, which enjoy the antibacterial properties and can attenuate the sun’s ultraviolet rays, appeal to the pharmaceutical and cosmetic industries^8,9^.

Another group of nanoparticles, which has stimulated the researchers to pay special attention to in recent years, involves carbon nanotubes. Carbon nanotubes (CNTs) are among the nano-carbon structures, featuring such unique characteristics as the high length-to-diameter ratio, high specific surface, excellent chemical stability, and so on^10,11^. Carbon Nanotubes, due to their hollow and small structure, have made huge progress in the fields of medicine and pharmaceuticals^12^.

The carbon nanotubes are taken advantage of in different processes, for example, the transfer and gene expression, targeted transfer of medical agents into cells, help in quick identifying pathogens, and measurement of blood glucose. The main reason for selecting carbon nanotubes in medical and pharmaceutical practice is their ability to absorb or bind themselves to a wide range of biomolecules^13^. However, one of the weak points of carbon nanotubes is that their lateral surfaces are so hydrophobic that they are usually accumulated in clusters. This issue restricts their application in biological environments to some extent. Therefore, in order to increase the activity of carbon nanotubes as well as their better dispersion in solvents, they are modified through various functional groups^14–16^. The chemical functionalization of carbon nanotubes can surprisingly alter their chemical and physical properties, and it leads to the modification and development of their functions in biological operations^14^. Some benefits of the functionalization of CNTs are as follows: Maintaining the stability of CNTs in the presence of large amounts of salt; better biocompatibility; maintaining the ability of the functionalized CNTs to pass through a cell membrane; the capability of secondary binding to medications; reducing the superficial hydrophobic area; decreasing the nonspecific adsorption of proteins by pharmaceutical carriers.

It is believed that these functionalized CNTs are suitable candidates to improve the partition coefficient of biomolecules in ATPSs^16–19^. To the best of our knowledge, the potential of using nanoparticles to achieve an increase in the partitioning of biomolecules has been studied by a small number of researchers^4–6^. To give some examples, the effect of gold nanoparticles on the partitioning of horseradish peroxidase in an ATPS containing polymer and dextran has been investigated by Keating *et al*.^4^, and the role of silica nanoparticles in the partitioning of α-amylase enzyme in an ATPS comprising polymer and salt has been evaluated by Dehnavi *et al*.^5^; also, the way graphene nanoparticles, graphene oxide, titanium dioxide, and alumina impact on the partitioning of cephalexin antibiotic in an ATPS of polymer and salt has been the subject of a study carried out by Afzal *et al*.^6^. In order to consider the efficiency of the functionalized CNT based ATPSs, vanillin was selected as a prevalent flavor. Vanillin is widely consumed as a natural additive, and its usage in the food, pharmaceutical, cosmetic, and sanitary industries^20^ has been continuously growing. Apart from being a food and pharmaceutical substance^19,21–24^, vanillin is regarded as a bioactive compound exhibiting antioxidant, antimicrobial, anticancer, and antitumor characteristics. Also, vanillin (on account of its phenolic properties) serves as a chemical mediator in the production of medications and chemical substances^19^. The two main methods of achieving natural vanillin include (Ι) direct extraction of herbal resources, and (ΙΙ) microbial biotransformation reactions. The extraction of vanillin from herbal resources is a costly and no economical process^19,21–23^. Hence, the necessity of developing vanillin extraction process, which is in high demand, so that the use of cost-effective techniques, which are compatible with the environment, has a major significance^19^. The ATPS is one of the methods for separating vanillin from byproduct and other undesirable components in the downstream process.

In the last decade, the partitioning of vanillin in the ATPSs containing carbohydrates and ionic liquids has been taken into account^24^. The ionic liquids are expensive; besides, IL-based ATPSs, in contrast with typical ATPSs have complexities that limit their application. Some of these difficulties are the different effects of the anions and cations of ILs on the ionic strength, the hydrophobic effect of the system, and the complexity of understanding the different behaviors of ILs.

However, the main question which was proposed in this study is: Which is the best system for separating vanillin from the different ATPSs by other researchers? How is it possible to increase the mass transfer of vanillin from one phase to another phase? The consideration of the partition coefficient makes it possible to select the optimum system for the separation and purification of vanillin.

In this study, a different scenario has been conducted in order to boost the efficiency of the ATPS. In the first step, an attempt was made in this scenario, so that the high efficiency, high performance, selectivity, convenient recycling of the target biomolecule, low cost, and quick mass transfer can take top priority. Therefore, the capability of the CNTs and metal oxide nanoparticles in the ATPSs (composed of polyethylene glycol (PEG) and sodium sulfate, and also polyethylene glycol and dextran) for increasing the partitioning of vanillin was assessed. The study of different nanoparticles allowed us to gauge the potential of this ATPS as an alternative purification platform. The salting-out phenomenon of sodium sulfate salt in a selective ATPS (PEG + Na_2_SO_4_) was investigated with the aid of the non-random two-liquid models (NRTL) model, and then the ATPS was modified by tiny quantities of low cost, biocompatible functionalized multi-walled carbon nanotube (MWCNT). The second step in this scenario is the molecular understanding of the partitioning mechanism of vanillin as a phenolic compound in the MWCNTs-based ATPSs, and also systematic, a comprehensive study on a molecular scale.

## Results and Discussion

In conducting the experiments, a repeatable method was utilized to achieve the maximum partition coefficient of vanillin in the ATPS. The potential of biomolecule extraction in a normal ATPS can marvelously be modified by introducing an appropriate nanoparticle as the biomolecule carrier into the system.

Therefore, in this study, the experiments were designed for a five-step course of action: in the first step, the vanillin partition coefficient in the two different ATPSs (PEG4000 + Na_2_SO_4_ + H_2_O/PEG4000 + DEX 15000 + H_2_O) was determined in the absence of nanoparticles. In the second step, the NRTL thermodynamic model was employed to study the effect of salting-out extraction on the separation. In the third step, aiming to raise the partition coefficient of vanillin, the potentiality of multiple different metal oxides nanoparticle and the strength of various MWCNTs were demonstrated in small quantities of the nanoparticles (0.01 wt%) as additives. In the fourth step, for increasing the efficiency of ATPSs, the surface of MWCNT was tailored by attaching an appropriate functional group to it. In the fifth step, the best ATPS was picked out to investigate the molecular mechanism of the vanillin partitioning. This favored ATPS consisted of polymer and salt along with a functional nanoparticle, which was able to increase the partition coefficient of vanillin.

### Salting-out effect by the NRTL thermodynamic model

In this section, a predictive thermodynamic model, such as the NRTL model, was applied to examine the following: (1) Exploring the possibility of formation of aqueous two-phase systems without the use of experimental data. (2) Development of a simple and fast predictive model without the experimental data and using existing binary interaction data. (3) Predicting the salting-out effect. (4) Determine the two-phase region with the predictive model.

The salting-out effect of Na_2_SO_4_ in the {PEG4000 (A) + Na_2_SO_4_ (B) + H_2_O {ATPS is studied with the help of the activity of water. The salting-out effect is investigated by intermolecular forces between species in this system whose deviation from the ideal state can be found out using the following equations^25^.

1 $$({a}_{w}+1)-({a}_{wA}^{o}+{a}_{wB\,}^{o})=0$$

Or

2 $$\Delta P-(\Delta {P}_{A}^{o}+\Delta {P}_{B\,}^{o})=0$$

wherein Eq. (1)., ${a}_{wA}^{o}$ and ${a}_{wB}^{o}$ are the activity of water in {A + water} and the activity of water in {B + Water} binary solutions; similarly, ${a}_{w}$ is the activity of water in {A + B + water} ternary system with the same molalities as those in the corresponding binary solutions. Also, $\Delta P={\rm{P}}-{{\rm{P}}}^{\ast }$ and ${\Delta P}_{{\rm{i}}}^{0}={{\rm{P}}}_{{\rm{i}}}^{0}-{{\rm{P}}}^{\ast }\,({\rm{i}}={\rm{A}},B)$, are the depressions of pressure in the ternary and binary solutions, respectively. Equations (1) and (2) are valid for semi-ideal solutions (SIS). In SIS, the interaction between A-B is not considered. Thus, if $({a}_{w}+1)-({a}_{wA}^{o}+{a}_{wB\,}^{o})$ or $\Delta P-(\Delta {P}_{A}^{o}+\Delta {P}_{B}^{o})$ deviate from zero (i.e. non-ideal solution), the interactions between (A-B) are significant. In the case of ternary solutions, if the interactions between (A-B) are more prevalent than those between (A-water) and (B-water), the salting-in phenomenon takes place, which can be expressed as^25^:

3 $$({a}_{w}+1)-({a}_{wA}^{o}+{a}_{wB}^{o}) > 0$$

4 $$\Delta P-(\Delta {P}_{A}^{o}+\Delta {P}_{B\,}^{o}) > 0$$

In the salting-in phenomenon, the mutual solubility of A-water and B-water increases. On the other hand, if the interaction between (A-B) is less prevalent than the interactions between (A-water) and (B-water), the salting-out phenomenon occurs, which means:

5 $$({a}_{w}+1)-({a}_{wA}^{o}+{a}_{wB}^{o}) < 0$$

6 $$\Delta P-(\Delta {P}_{A}^{o}+\Delta {P}_{B\,}^{o}) < 0$$

When it comes to the salting-out phenomenon, the interaction between A and B is unfavorable, and the species A and B repel each other. As a result, in different concentrations of species A and B, the aqueous biphasic systems can be formed thanks to the entropic effects. The NRTL model has been applied for predicting the activity of water in (A + water) and (B + water) aqueous binary solutions and their ternary systems. The binary interaction parameters of the NRTL model between (A-water), (B-water) and (A-B) pairs were obtained from the literature^26^. In this model, it is assumed that the salt cannot be dissociated into ionic species^26,27^.

**Figure 1:**
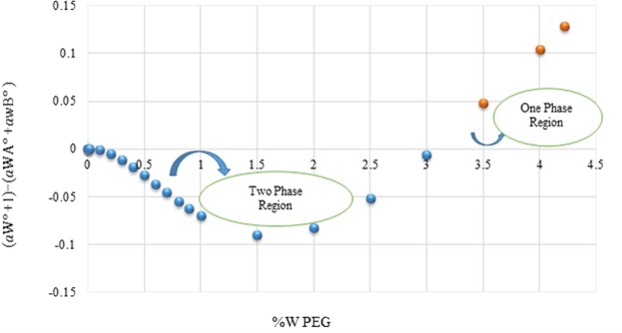
Plot of $({a}_{w}+1)-({a}_{wA}^{o}+{a}_{wB}^{o})$ the verses weight fraction of polyethylene glycol in aqueous solution.

Variations of $({a}_{w}+1)-({a}_{wA}^{o}+{a}_{wB}^{o})$ for the polymer molality is shown in Fig. 1.

According to Fig. 1 at low concentrations of polymer, the deviation from the ideal state is negative, and the system is bound to form an aqueous two-phase system. In this state, the salting-out phenomenon prevails. Whereas, at higher concentrations of polymer, the deviation from the ideal state is positive, and the salting-in phenomenon occurs. It can be concluded that the interaction between the polymer and salt is less favorable than the interactions between the salt and water, as well as the polymer and water, leading to the formation of a biphasic system.

### Effect of nanoparticles on the partitioning of vanillin

The effect of adding kinds of mineral and organic nanoparticles on the partition coefficient of vanillin (K_van_) in two different ATPSs (PEG4000 + Na_2_SO_4_ + H_2_O/PEG4000 + DEX 15000 + H_2_O) has been reported in Table 1. As it is clear from Table 1, the partition coefficient of vanillin is higher than 1, which indicates that vanillin tends to migrate toward the top phase. This preferential migration has appropriate accordance with the octanol-water partition coefficient of vanillin ($log{K}_{ow}=1.19$)^28^. It means that the vanillin is inclined to migrate toward the phases, which are more hydrophobic^28^. By observing the partition coefficients of these two ATPSs (PEG4000 + Na_2_SO_4_ + H_2_O/PEG4000 + DEX 15000 + H_2_O), it can be deduced that the ATPS containing polymer and salt has a more significant impact on the improvement of the partition coefficient of vanillin. Consequently, the successful enhancement of the extraction process depends mainly on the two adjacent phases. The sodium sulfate salt has a positive impact on the partitioning of vanillin, and this is due to a potent salting-out effect induced by $S{O}_{4}^{2}$^−^. The data in Table 1 indicates that the partition coefficients of vanillin have diminished in the presence of the Multi-walled carbon nanotubes (MWCNTs) and single-walled carbon nanotubes (SWCNTs). MWCNTs are insoluble in aqueous systems and have weak chemical compatibility with polymer compounds. The previous research studies have suggested that the superficial modification of MWCNTs causes their compatibility to improve as well as their solubility to increase^27^. Therefore, surface modification of MWCNT by a functional group compatible with the biomolecule can be a significant factor in enhancing the partition coefficient.

**Table 1:** Effect of Nanoparticle Additives on Partitioning of Vanillin.

System	K ± σ^a^	System	K ± σ^a^
PEG + Na_2_SO_4_	19.260 ± 0.393	PEG + DEX	1.384 ± 0.062
PEG + Na_2_SO_4_ + Funct.MWCNT^b^	43.730 ± 0.087	PEG + DEX + Funct.MWCNT^b^	1.988 ± 0.09
PEG + Na_2_SO_4_ + SWCNTs	4.123 ± 0.098	PEG + DEX + SWCNTs	1.222 ± 0.100
PEG + Na_2_SO_4_ + MWCNTs	6.652 ± 0.658	PEG + DEX + MWCNTs	1.335 ± 0.111
PEG + Na_2_SO_4_ + n-ZnO	23.180 ± 0.197	PEG + DEX + n-ZnO	2.110 ± 0.221
PEG + Na_2_SO_4_ + n-Fe_2_O_3_	5.845 ± 0.142	PEG + DEX + n-Fe_2_O_3_	1.200 ± 0.080
PEG + Na_2_SO_4_ + n- TiO_2_	5.650 ± 0.658	PEG + DEX + n-TiO_2_	1.224 ± 0.123

^a^standard deviation; ^b^functionalized MWCNT.

### Surface modification of multi-walled carbon nanotubes

The stability and the decreased accumulation of carbon nanotubes in the aqueous and polymer solvents are regarded as influential factors when it comes to efficiency optimization. Surface modification of MWCNTs by the carboxyl group creates a negative charge on the MWCNT surface^17,27,29^. In order to investigate the presence of the –COOH and –OH functional groups on the nanotubes surface, the FTIR spectroscopy has been made use of in our study (Figs. S2 and S3). The absorption bands at 1460 and 1625 cm^−1^ are assigned to the C=C stretching vibration. Also, the stretching vibration at 3403 cm^−1^ is an evidence of the presence of –OH group (Fig. S2). The FTIR spectroscopy of modified MWCNTs shows that the intensity of the C=C stretching vibrations has declined, and this means that the bonds have changed. On the other hand, several vibrations have been observed at 1500 cm^−1^ and 1000–1200 cm^−1^, which are related to the O–C=O and C–O groups available in carboxylic acid, respectively. The presence of the –COOH group was confirmed at the 724–940 cm^−1^ region. These results imply the presence of –COOH and –OH groups on the surfaces of MWCNTs (Figs. S2 and S3).

FESEM is an essential tool to characterize the direct observation of the size, shape, and structure of any nanomaterial. The FESEM image of the modified multi-wall carbon nanotubes demonstrates that they were not damaged after the functionalization processes since they had a uniform morphology. Nevertheless, the ends of carbon nanotubes have converted to amorphous carbon under the acidification conditions, which opened the two ends of MWCNT owing to the presence of the pentagon rings and the local strains (which is attributed to the shape of a pyramid). As a result, the end of MWCNT has higher reactivity than its wall. Nonetheless, by replacing the carboxyl group, the diameter of the MWCNT grows, which verifies that the –COOH and –OH group has successfully been attached to the MWCNT surfaces. Interestingly, these results agree with those reported by other researchers^15,30,31^ (Figs. S4 and S5).

**Figure 2:**
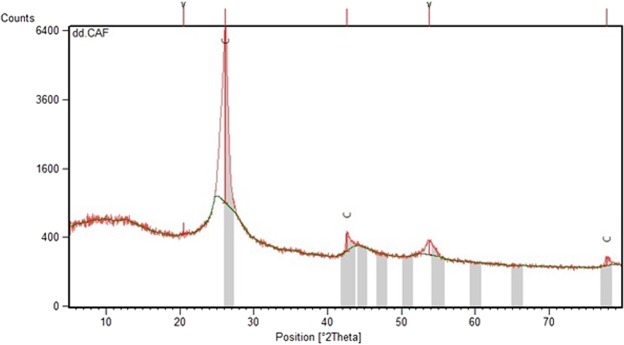
X-ray diffraction analysis (XRD) for functionalized MWCNTs.

The structural property of the modification of MWCNTs was investigated by XRD (at an excitation wavelength of 0.154 nm), as shown in Fig. 2. The diffraction angle of the C (002) peak for the graphite is located at 26.174°. The sharpness of this peak indicates the MWCNTs’ graphite structure and the absence of noticeable deficiencies.

The analysis of x-ray diffraction profiles is the usual and standard way to measure the crystallite size of nanocrystals. The crystallite size (D) was obtained by applying the Scherrer relation as follows^32^:

7 $${\rm{D}}=\frac{0.9{\rm{\lambda }}}{{\rm{\beta }}\,\cos \,{\rm{\theta }}}$$

where λ is the synchrotron radiation wavelength 0.154 nm, θ is the position of the (100) peak,

and β (0.357°) is the half-height width of the (100) peak of graphite in 2θ (rad) units. The average size of the functionalized MWCNT crystallites was estimated at 26 nm according to the Scherrer relation.

### Improved partitioning mechanism using functionalized MWCNT

The experimental data of the partition coefficient showed that the modification of the MWCNTs surface with the carboxyl functional group was a tremendous success in refining the separation of vanillin in an ATPS, in a way that an outstanding value of the vanillin partition coefficient (*i.e*. K = 43.73) in the ATPS containing carbon nanotubes modified with the carboxyl groups was attained at the temperature of 25 °C and atmospheric pressure. Among the ATPSs presented in Table 1, the {PEG + Na_2_SO_4_ + functionalized MWCNT} system has had an exceptional surge in the partition coefficient of vanillin. Accordingly, by adding the amount of 0.01 wt% of functionalized MWCNT to the ATPS, the partition coefficient has climbed almost 127 percent compared to the corresponding system without nanoparticle.

**Figure 3:**
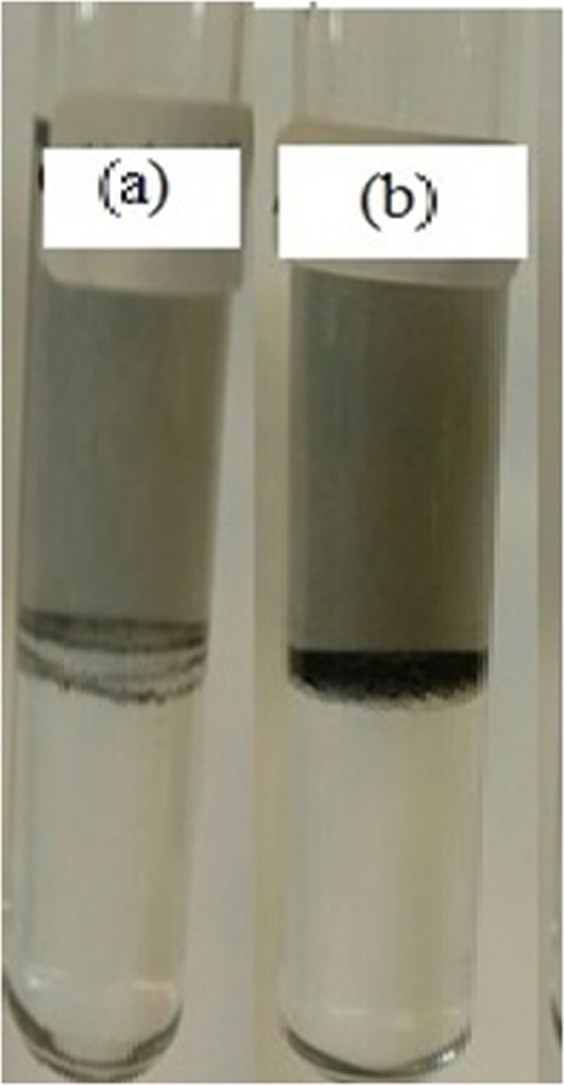
The photographs of the vanillin partitioning in two different ATPSs: (**a**) PEG + Na_2_SO_4_ + H_2_O + Vanillin + MWNTs; (**b**) PEG + Na_2_SO_4_ + H_2_O + Vanillin + functionalized MWCNT.

The higher partition coefficients, obtained from incorporating functionalized MWCNT into a single-stage ATPS process, suggest the substantial contribution of these nanoparticles promising substitutes toward enhancing the traditional extraction methods, and, for this reason, they should be further brought into focus in the biotechnology areas. The photographs of the vanillin partitioning in two different ATPSs have been illustrated in Fig. 3. These photographs indicate the visual assessment related to the stability of each system involving nanoparticle. As shown in Fig. 3, the dispersion and distribution of MWCNTs are lower than those of functionalized MWCNT, which can be ascribed to the hydrophobic properties, insolubility, low functional groups, and low Zeta potential of the MWCNTs^29,31,33^. Moreover, the MWCNT tends to form agglomerates, so the MWCNT moves toward the bottom phase^33^. This phenomenon implies a reduction in the partition coefficient. Figure 3 shows that functionalized MWCNT have completely migrated toward the top phase.

The specific surface area of MWCNT increases after modification with -COOH. These functional groups attached on the surfaces of the MWCNT increase the partition coefficient of vanillin due to the improved surface area and specific interaction vanillin with the -COOH groups. These functional groups increase the vanillin partition coefficient by the effect of two factors simultaneously. The effect of these factors simultaneously is more effective rather than separately.

Some researchers have reported that a higher net surface charge and a zeta potential of greater than │−15 mV│ having are factors that prevent the functionalized MWCNTs from accumulating and agglomerating in aqueous solutions^33^. As maintained by the stability theory, in emulsions with high zeta potential value (negative or positive), the electrostatic repulsion between the particles increases, leading to the nanoparticle stability in the emulsion.

On the other hand, *van der Waals* forces drop as a result of bonding the carboxyl and hydroxyl groups on the surface of carbon nanotubes. In this way, the separation and dispersion of the carbon nanotubes in water become better, and also the sedimentation and accumulation processes will not take place. The hydroxyl and carboxyl groups available on the surface of the modified carbon nanotubes break down into the water, and these groups create a negative charge on the surface of the nanotubes. This phenomenon can give rise to a growth in the partition coefficient of vanillin. Photographs related to the stability and dispersion of the MWCNTs and functionalized MWCNT in pure water can be found in the Supporting Information (Fig. S6).

### The impact of functionalized MWCNT on the partitioning mechanism of vanillin

In order to investigate the mechanism of the increased partition coefficient of vanillin in the target ATPS (PEG4000 + Na_2_SO_4_ + Vanillin + functionalized MWCNT), a systematic study was performed on the top phase. The FT-IR spectrum has been turned to account in the inspection of the structure and interactions between vanillin and the functional groups of MWCNTs.

**Figure 4:**
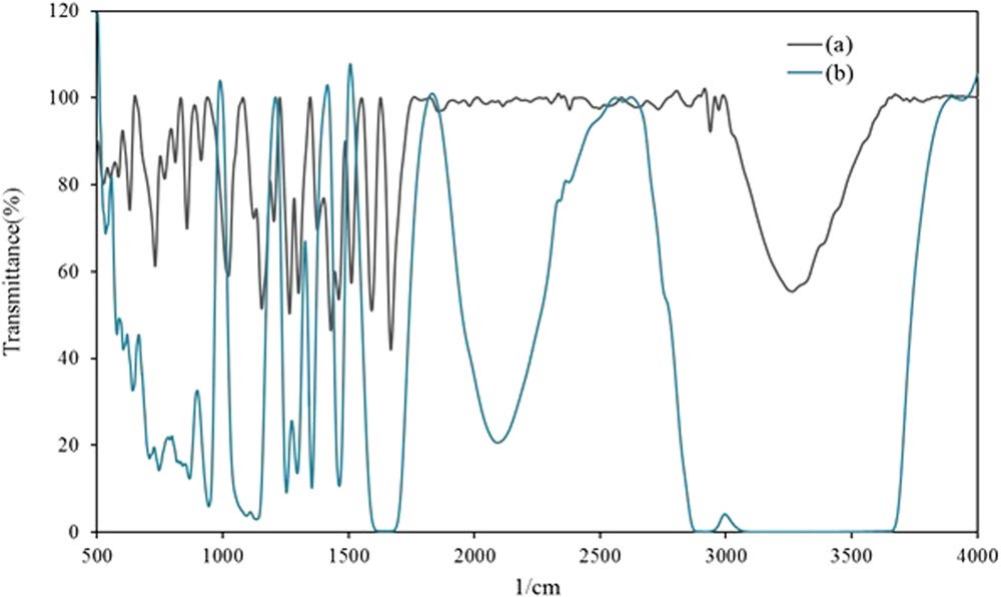
FTIR spectra of top phase of two ATPS systems: (**a**) PEG+Na_2_SO_4_+H_2_O+Vanillin; (**b**) PEG + Na_2_SO_4_ + H_2_O + Vanillin + functionalized MWCNT.

The reason for the increase in the vanillin partition coefficient is the formation of hydrogen bonds between vanillin and functionalized MWCNT. The FT-IR spectra for the top phases of two different ATPSs (PEG4000 + Na_2_SO_4_ + H_2_O + Vanillin; PEG4000 + Na_2_SO_4_ + H_2_O + Vanillin + functionalized MWCNT) have been displayed in Fig. 4.

The functional groups available in the system are as follows: –OH, –COOH and –COO^−^. With reference to Fig. 4, the wavelength range 3400–3600 cm^−1^ is assigned to the –OH stretching vibration, whereas the wavelength range 724–940 cm^−1^ is associated with the –COOH stretching vibration, and the wavelength range 1400–1407 cm^−1^ corresponds to the –COO^−^ stretching vibration. These functional groups facilitate the development of hydrogen bonds between vanillin and functionalized MWCNT, resulting in a more efficient vanillin extraction. The FTIR spectra of pure substances (Vanillin and functionalized MWCNT) are available in Figs. S2 and S7.

**Figure 5:**
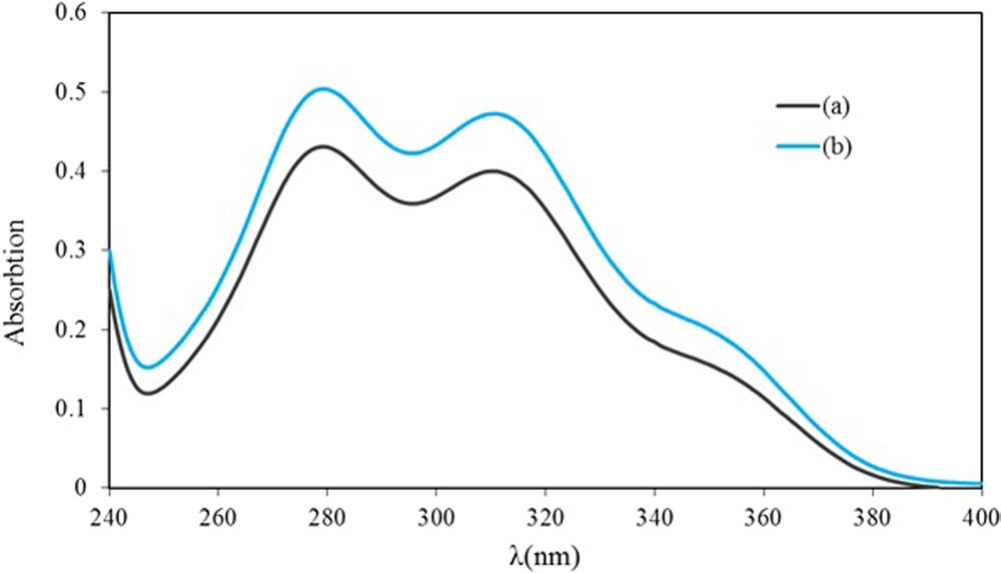
Maximum of vanillin absorption in the top phase (polymer-rich) (**b**) the presence and **(a**) absence of modified carbon nanotubes.

The UV-vis spectra helps to examine the conformation of vanillin in the top phase of the ATPS^28^. First, the maximum absorption of vanillin was determined in distilled water, and afterward, the maximum absorption of vanillin was evaluated in the polymer-rich phase in the presence and absence of nanotubes. The results obtained from the UV-vis spectra have been reported in Fig. 5. The comparison among the spectra revealed that the maximum absorption of vanillin (at the wavelength of 280 nm) was the same in all of them^28^. This means that there are no direct chemical bonds interacting between vanillin and the PEG molecules in the extraction process, confirming no distortion in the spatial structure of the vanillin.

**Figure 6:**
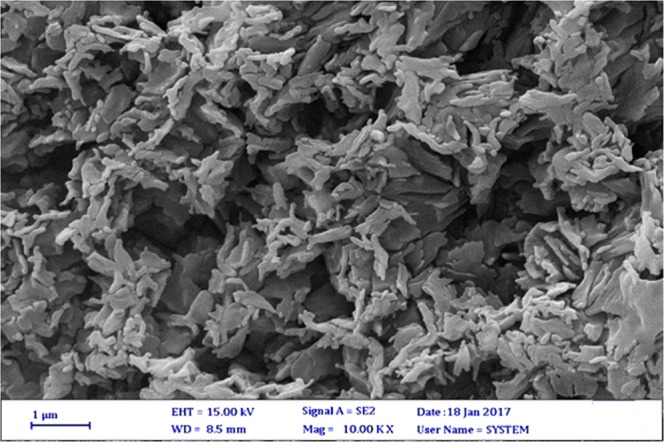
FE-SEM image for top phase without nanoparticle.

**Figure 7:**
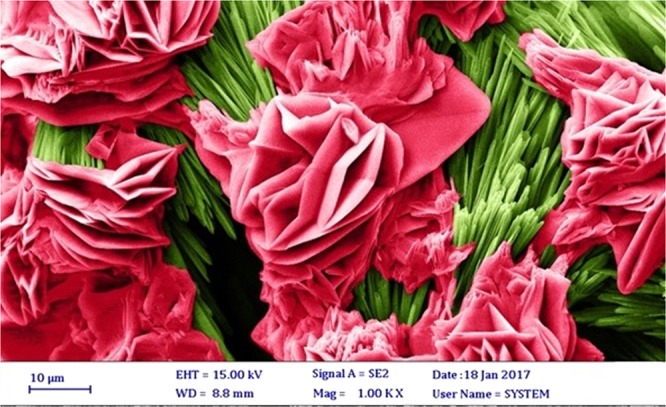
FE-SEM image for top phase with the nanoparticle. The red color represents vanillin + PEG and the green color represents functionalized MWCNT.

The microscopic structure of the PEG-rich top phase is detected with the help of the FESEM for a deeper understanding of the separation process. Figure 6 illustrates the conformation of the PEG-rich top phase without nanoparticle. There are no nanoparticles in this Fig. The particles of polyethylene glycol and vanillin have been agglomerated. Figure 7 shows the FESEM image of the top phase (PEG-rich) after the addition of the functionalized MWCNT to the system. The tendency of vanillin and polyethylene glycol towards nanotubes are visible, as well. XRD analysis helps us to roughly calculate the average size of the functionalized MWCNT crystallites as 26 nm. According to the FESEM image of the top phase, the size of particles in the top phase rose to over 500 nm, implying the formation of hydrogen bonds between the vanillin and MWCNTs. The functional groups in the functionalized MWCNT with the electronegative atom facilitate the development of hydrogen bonds between vanillin and functionalized MWCNT.

Consequently, a Comparison between the two systems (ATPS and ATPS + nanoparticles) indicates that a higher amount of vanillin is disposed to shift from the hydrophilic phase (Na_2_SO_4_-rich) into the hydrophobic phase (PEG- rich) in the system containing nanoparticles.

The instability of particles in the top phase of ATPS is likely to lessen the vanillin partition coefficient. The zeta potential value of the top phase (*i.e*. 12.4 mv) proves the relative stability of the system. The hydrogen bond between the molecule of functionalized MWCNT and the vanillin molecule can justify this stability.

**Figure 8:**
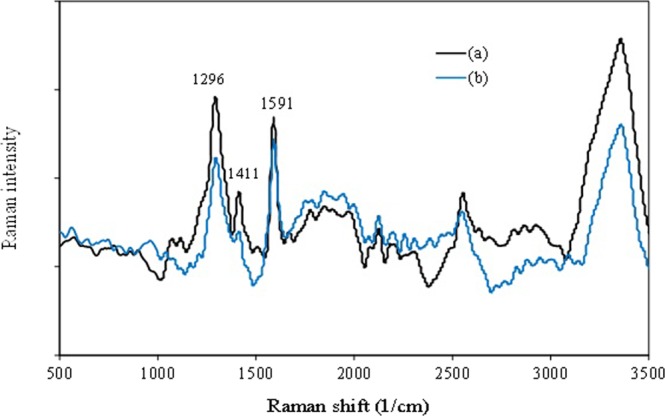
Raman spectra of (**a**) PEG + Na_2_SO_4_ + functionalized MWCNT and **(b**) PEG + Na_2_SO_4_ + functionalized MWCNT + vanillin.

Raman scattering spectroscopy was performed to investigate the structural properties of functionalized MWCNT^34,35^. Figure 8 shows the Raman scattering spectroscopy using a 633 nm Helium-Neon laser. Raman scattering spectroscopy was performed to study the structural alterations of the functionalized MWCNTs in the top phase of the ATPS made up of PEG + Na_2_SO_4_ + H_2_O + functionalized MWCNT and PEG + Na_2_SO_4_ + H_2_O + functionalized MWCNT + Vanillin.

Two Raman peaks at the wavenumbers of 1591 and 1296 cm^−1^ correlate with the G-band (which is attributed to the graphene bond) and the D-band (stemming from the disorderliness and structural deficiencies) of functionalized MWCNT, respectively^36,37^. These peaks are characteristic of CNTs^35,37^. The sharp and intense single-crystallite graphite G-band is located around 1591 cm^−1^ and is induced by the bond stretching of the carbon atom’s sp^2^ orbital electron in the rings and chains. The graphitization is characterized by a wavenumber shift and a line width broadening of the G-band as well as the peak intensity ratio of the D-band to the G-band (I_D_/I_G_) signal^35,37^. An increase in this ratio (I_D_/I_G_) means the destruction of a part of functionalized MWCNT.

As it is apparent from comparing (Fig. 8a,b), the intensity of the peak representing structural deficiencies has experienced an augmentation. This increase indicates the successful placement of vanillin particles on functionalized MWCNT. The spectrum in the curve a, b exhibits two intense bands at 1591 and 1296 cm^−1^, which correspond to ν (C=C) and δ (O-H), respectively. The band at 1411 cm^−1^ is assigned to δ_ad_ (CH_3_), although some contribution from ring stretching is also possible in the region of 1000–1100 cm^−1^. These peaks also are seen in the curve a, while their intensity is high. The weak and medium bands appearing in the 2918 and 2858 cm^−1^ are associated with the ν_as_ (CH_3_) and ν (CH) of the aldehyde functional group.

## Conclusions

This piece of research has demonstrated that modified MWCNTs have the potential for augmenting the partition coefficient of a phenolic compound like vanillin by 127 percent. MWCNTs assist the extraction by adsorbing Vanillin and thus allowing the remaining Vanillin to partition according to the LL-partition coefficient. Our findings suggest that the ATPSs incorporating the functionalized MWCNTs can be a viable alternative to the conventional extraction techniques. This approach is cheaper, simpler, and not to mention more efficient in comparison with IL-based ATPSs. An in-depth analysis of the molecular mechanism behind the partitioning of vanillin has revealed that, with a slight methodical manipulation of typical ATPSs (for example, by inserting proper nanoparticles in the system), an ideal extraction of vanillin into the top phase of ATPS through a single-step operation is readily attainable. The achievement of this work, besides the previous successful drug delivery studies employing carbon nanotubes as carriers, can open up a unique opportunity to perform the whole process of synthesis and separation of the MWCNTs/vanillin complex used in medical treatment. The product can be isolated by filtration of the ATPS top phase.

## Methods

### Experimental materials

Polyethylene glycol (PEG) was supplied by Merck (Darmstadt, Germany) with the average molecular weight (MW) of 4000 g.mol^−1^ and with a purity of 99%. Sodium sulfate (>99%) was purchased from Merck (Darmstadt, Germany). Sodium sulfate was selected concerning the desired pH (pH = 6.5–7) for vanillin extraction. Vanillin (4-hydroxy-3-methoxy benzaldehyde) with a purity of 99% and dextran (MW = 15000, Purity> 99%) were procured from Sigma-Aldrich (St. Louis, MO). Multi-wall carbon nanotube (outer diameter = 10–20 nm, inner diameter = 5–10 nm, maximum length = 30 µm, Purity>95%) was provided by Neutrino company (Tehran, Iran). Sulfuric acid (H_2_SO_4_, Purity> 99%) and nitric acid (HNO_3_, Purity> 99% wt) were obtained from Merck (Darmstadt, Germany) for the functionalization of carbon nanotubes. *Zinc oxide nanoparticles* (n-*ZnO*, Purity> 99%) *(*US Research Nanomaterials, Inc*)*, single-walled Carbon Nanotubes (n-SWCNT, Purity>95%) *(*US Research Nanomaterials, Inc*)*, *titanium dioxide nanoparticles (n-TiO*_*2*_, Purity> 99%) *(Aeroxide P25, Degussa Evonik), Iron oxide nanoparticle* (n-Fe_2_O_3_, Purity>95%wt) *(*US Research Nanomaterials, Inc*)* were used as received. Distilled water was produced by the laboratory equipment (RO-LAB, DW65), applying twice distillation reverse osmosis.

### Method

The concentration of the components of the two phases in equilibrium (working point) (20% PEG + 10% Salt + 70% H_2_O) in the two-phase region was determined based on our previous experiences of the polyethylene glycol/sodium sulfate ATPS^28^. The concentration of the components in the two-phase region (8.5% PEG + 6% DEX + 85.5% H_2_O) was selected regarding the phase diagram reported in the Supporting Information (Fig. S1). According to our previous experimentation and respecting the standards (like the possibility of the easy separation of two phases, simple sampling, and the equal volumes of the top and bottom phases), the working points within the liquid-liquid area were chosen. All the concentrations of the constituents utilized in the system have been calculated on the basis of mass percentage^28,38^.

In order to purify the target biomolecule in the ATPSs containing nanoparticles and without nanoparticles, the details of the vanillin partitioning process has been given in the Supporting Information. The absorbance of vanillin in the top and bottom phases were determined through spectrophotometry (UV/vis Model: sp-2100uv, USA) at the wavelength of 280 nm where the maximum absorption of vanillin occurred^20^.

In this work, the carbon nanotubes were treated by acid mixture (HNO_3_/H_2_SO_4_) to attach the carboxyl group (COOH) and the hydroxyl group (OH) to the surface of MWCNTs. The procedure of the superficial modification of carbon nanotubes has been described in the Supporting Information. The recognition of the chemical structures of the compounds and functional groups was performed using Perkin – Elmer Fourier Transform infrared spectroscopy (FT-IR spectroscopy) at the ambient temperature. The FT-IR spectra of the pure vanillin, pure carbon nanotubes, and functionalized MWCNTs in the top phase of ATPSs were recorded in the wavelength range 400–4000 cm^−1^. Thermo Nicolet FT-Raman 960 spectrometer was utilized for more accurate identification of the functional groups. The Zeta potential of the nanoparticles was done via a Nano ZS *(*red badge*)* ZEN 3600 device from Malvern Co. The microscopic structure of the pure carbon nanotubes and modified MWCNTs in the top phase of ATPSs was detected by the field emission scanning electron microscopy (FESEM, MIRA3TESCAN-XMU). The modified MWCNTs were characterized by X-ray diffraction (XRD) (Panalytical X’Pert Pro).

## Supplementary information

Supplementary Information
