# Supplementary material for: Increase of vanillin partitioning using aqueous two phase system with promising nanoparticles
Source: Sci Rep. 2019 Dec 23;9:19665. doi: 10.1038/s41598-019-56120-8 (PMC6927956; doi:10.1038/s41598-019-56120-8)
Supplement: Supplementary file 1 — Supplementary Information [file 41598_2019_56120_MOESM1_ESM.docx]

**Supplementary Information**

**Increase of vanillin partitioning using aqueous two phase system with promising nanoparticles**

Mitra Nouri ^a^, Shahla Shahriari ^b,*^, Gholamreza Pazuki ^c^

*^a^ Department of Food Science and Technology, Shahr-e-Qods Branch, Islamic Azad University, Tehran, Iran*

*^b^ Department of Chemical Engineering, Shahr-e-Qods Branch, Islamic Azad University, Tehran, Iran*

*^c^Department of Chemical Engineering, Amirkabir University of Technolgy (Tehran Polytechnic), Tehran, Iran*

Corresponding author.

*E-mail address:* [Shahla_shahriari@yahoo.com](mailto:Shahla_shahriari@yahoo.com) (Sh.Shahriari).

**Supplemental Methods**

**Drawing binodal Curves.** The binodal curves were determined at 298 K through the cloud point method, known as the visual determination technique. The cloud point titration method was used to draw the ternary phase diagrams with the polyethylene glycol (PEG) and dextran (DEX). The experimental procedure adopted has been validated in previous works ^1,2^.

At first, aqueous stock solutions of a known concentration of PEG (≈ 75wt% ) and DEX (at ≈ 2wt% ) were prepared. After that, a DEX solution was being added dropwise to the PEG solution (or vice versa) until the solution became cloudy, indicating an aqueous biphasic system. Dropwise addition was carried out under constant stirring and atmospheric pressure. Therefore, double distilled water was added dropwise so that the solution became clear and a monophasic region was observed. In the next step, the composition of the mixture was going to be specified. For this purpose, with the knowledge of the initial mixture composition and the amount of added solution, the total system composition was calculated, yielding a point on the binodal curve. This procedure was repeated until the required points for the binodal curves were obtained. In all steps, the solution was mixed with a magnetic agitator. The mass fractions of the components of the ternary system were estimated with an accuracy of ±10^-4^ g by the weight of all the added components. The experimental phase diagram for the aqueous two-phase systems consisting of the PEG 4000 + DEX 15000+ H_2_O is presented in Fig .S1.

**Determination of the partition coefficient of vanillin.**The experimental procedure for the measurement of the biomolecule partition coefficients in ATPSs was described elsewhere ^3-5^.

Two different mixture compositions were used: (20% PEG + 10% Na_2_SO4 + 70% H_2_O, and 8.5% PEG + 6% DEX + 85.5% H_2_O).

A certain amount of vanillin (0.01 g) was added to each mentioned biphasic system. The partitioning of vanillin was examined (i) in the absence and (ii) in the presence of the nanoparticles. In order to investigate the extraction of vanillin in the ATPSs featuring nanoparticles, initially 0.001 g of the nanoparticles was introduced into 2 mL of double-distilled water, and then the mixture was sonicated for 1 h.

In the next stage, the nanoparticle suspension was added dropwise to the ATPS, which contained vanillin, while the mixture was being agitated by a magnetic stirrer.

The weight fractions were weighed using a digital scale (with an accuracy of ±10^-4^ g). The mixture then was stirred for 30 min, and afterward, the system was placed into an incubator (Memmert, Germany) for 24 h in order to reach equilibrium and achieve the complete partitioning of vanillin between the two phases. The incubator was utilized to control the temperature with an accuracy of ± 0.01˚C. As the complete separation of the two phases was accomplished, the top and bottom phases were isolated using a plastic syringe, and they were poured into separate containers. The weight fractions of vanillin in the top and bottom phases were determined through spectrophotometry (UV/vis Model: sp-2100uv, USA) at the wavelength of 280 nm where the maximum absorption of vanillin occurred.

The experiments were conducted in a pH range of 6.5-7. The optimum pH value for vanillin is within, and it is worth noting that the sodium sulfate salt was chosen so that the biphasic systems could enjoy the appropriate pH values. The pH of each aqueous phase was determined at 25 (± 1) °C using a Microprocessor pH meter-206 (Lutron).

**Surface modification of carbon nanotubes.** For the purpose of modifying the surface of CNTs by oxygen functional groups, a pickling process was applied ^6,7^. In this technique, 0.1 g of MWCNTs were added to a solution of sulfuric acid / nitric acid (99%) with a ratio of 3:1. The mixture then was put into an ultrasonic bath (DSA100-SK2-4.0L, made by Universal Co.) for 4 h at room temperature so that the MWCNTs could be finely dispersed throughout the solution without being accumulated in some areas. The obtained suspension was filtered and, in turn, rinsed with distilled water several times. After the pH of the distilled water effluent had remained unchanged at about 7, the MWCNTs were dried in a vacuum oven for 24 h and at a temperature of 40 °C.

**Supplemental References**

1. Long, M.S., Keating, C.D., Nanoparticle conjugation increases protein partitioning in aqueous two-phase systems, *Analytical Chemistry*. **78**, 379-386(2006)
2. Dehnavi, S.M., Pazuki, Gh.R., & Vossoughi, M. PEGylated silica-enzyme nanoconjugates: a new frontier in large scale separation of α-amylase.*Scientific Reports*. **5**,1-7(2015)
3. Afzal Shoushtari, B ., Rahbar Shahrouzi, J., Pazuki, Gh.R. Effect of nanoparticle additives on partitioning of cephalexin in aqueous two-phase systems containing Poly(ethylene glycol) and Organic Salts.  *Journal of Chemical & Engineering Data*. **61**, 2605-2613 (2016).
4. Ebrahimi, T., Shahriari, Sh. Extraction of betanin using aqueous two-phase systems. *Bulletin of the Chemical Society of Japan*. **89**, 565-572(2016)
5. Jiang, X. The influence of acid treatment on multi-wall carbon nanotubes. *Pigment & Resin Technology*. **38**, 165-173(2009)
6. Li, Zh ., Fan, L., Zhang, T., Li, K. Facile synthesis of Ag nanoparticles supported on MWNTs with favorable stability and their bacterial properties. *Journal of Hazardous Materials*. **187** ,466- 472(2011)

**Supplemental Figure Legends**

**Figure S1.** Binodal curves for the ternary system with the PEG (1) + DEX 15000(2) + H_2_O (3) ATPs at 298 K.

**Figure S2.** FTIR spectra of functionalized MWCNT.

**Figure S3.** FTIR spectra of MWCNT.

**Figure S4.** FE-SEM image of MWCNT.

**Figure S5.** FE-SEM image of functionalized MWCNT.

**Figure S6.** Photographs of the stability and dispersion of nanotubes suspensions (a) MWCNTs suspensions and (b) functionalized MWCNTs suspensions.

**Figure S7.** FTIR spectra of vanillin.

**Figure S1.** Binodal curves for the ternary system with the PEG (1) + DEX 15000(2) + H_2_O (3) ATPs at 298 K.

**
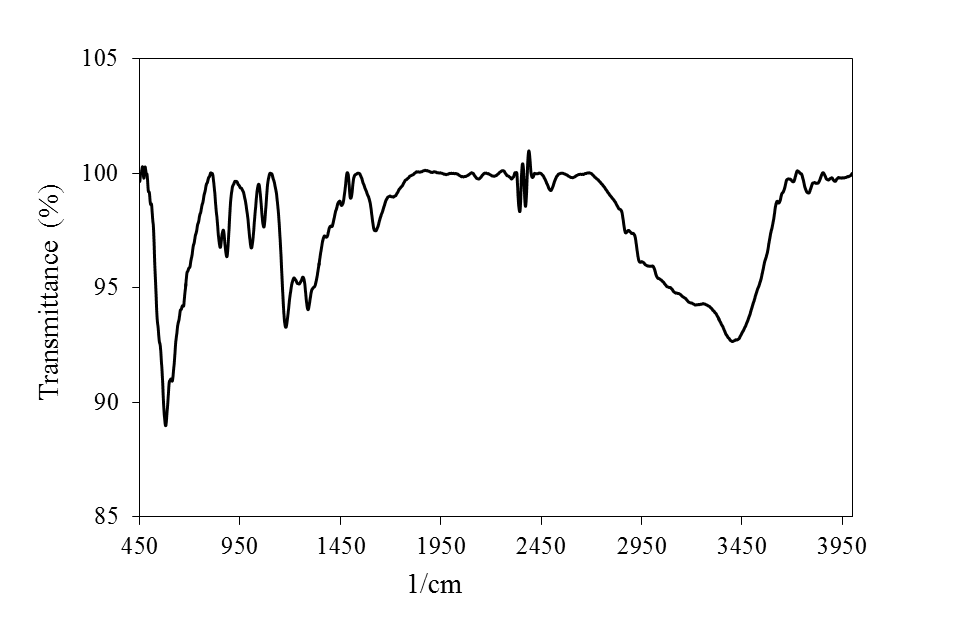
Figure S2.** FTIR spectra of functionalized MWCNT.

**Figure S3.** FTIR spectra of MWCNT.


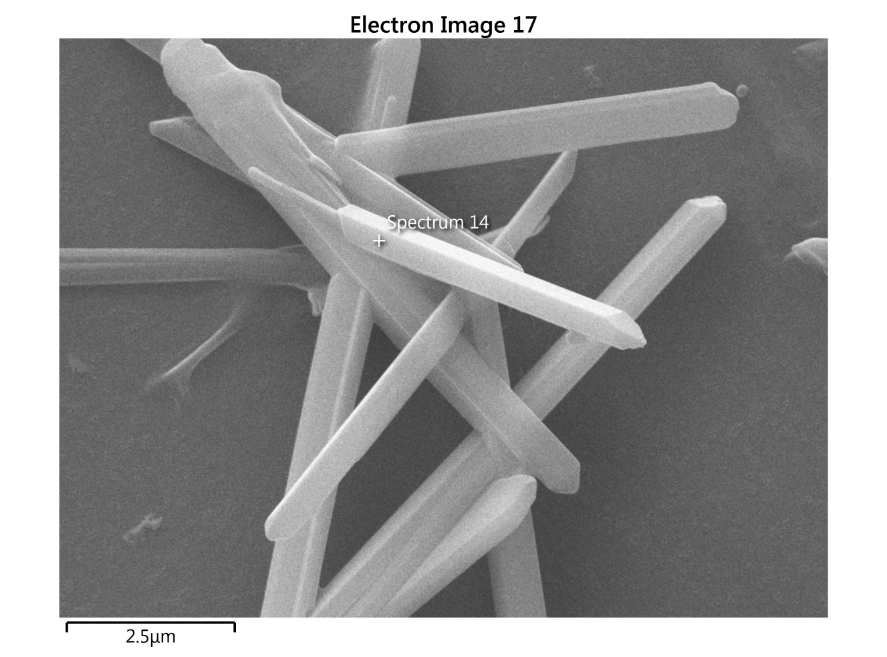
**Figure S4.** FE-SEM image of MWCNT.


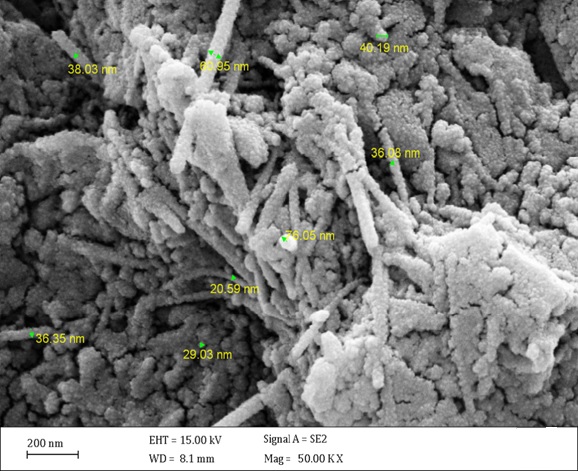


**Figure S5.** FE-SEM image of functionalized MWCNT.


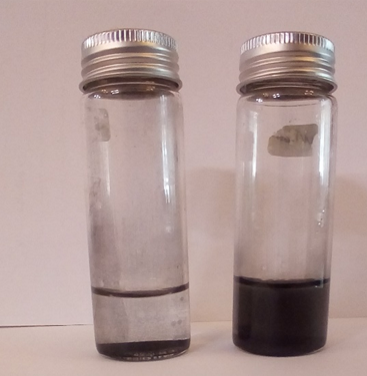


(a)

(b)

**Figure S6.** Photographs of the stability and dispersion of nanotubes suspensions (a) MWCNTs suspensions and (b) functionalized MWCNTs suspensions.


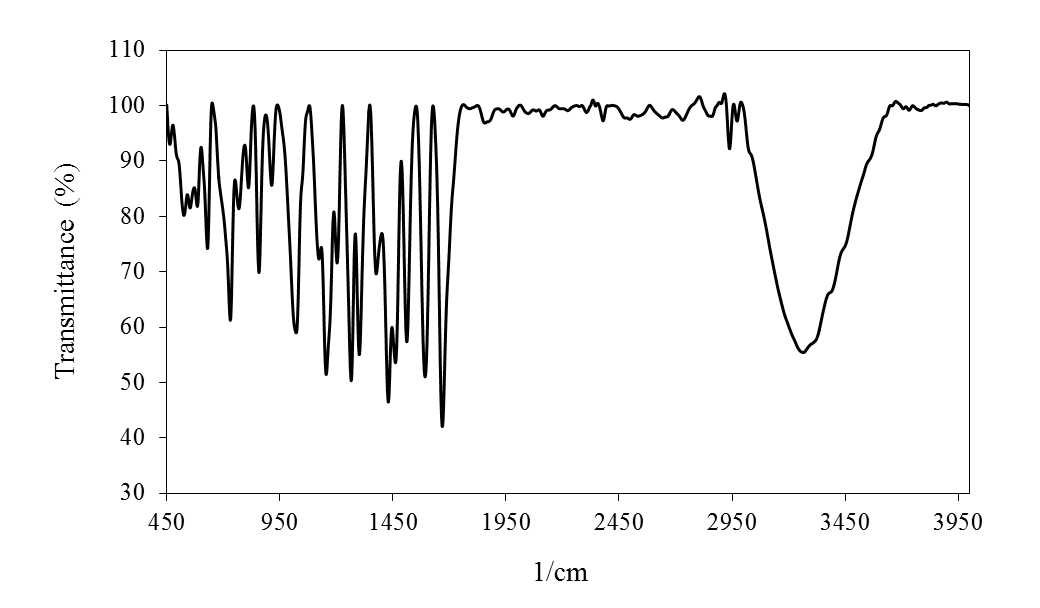
**Figure S7.** FTIR spectra of vanillin.
